# Patients Who Refuse Blood Transfusions Should Not Be Discouraged from Immediate Direct-to-Implant Breast Reconstruction After Mastectomy: A Retrospective Study on Severe Bleeding Risks

**DOI:** 10.3390/cancers17193137

**Published:** 2025-09-26

**Authors:** Maria Skonieczna, Piotr Strzałka, Marek Zadrożny, Wojciech Grabczak, Piotr Pluta

**Affiliations:** 1 Department of Surgical Oncology and Breast Diseases, Polish Mother’s Memorial Hospital—Research Institute, 93-338 Lodz, Poland; 2 Department of Hematology, Medical University of Lodz, 93-510 Lodz, Poland; 3 Department of Hematology and Transplantology, Multidisciplinary Provincial Centre of Traumatology and Oncology Nicolas Copernicus in Lodz, 93-513 Lodz, Poland

**Keywords:** mastectomy, immediate breast reconstruction, breast implant, bleeding, bloodless medicine patients

## Abstract

Background: Direct-to-implant breast reconstruction is the most common method for immediate breast restoration after mastectomies performed with prophylactic or curative indications. This approach significantly enhances patients’ quality of life without adversely affecting oncological outcomes. However, it remains unclear whether mastectomy with direct-to-implant breast reconstruction (M-DTI) is an optimal option for patients who refuse blood transfusions for religious reasons (e.g., Jehovah’s Witnesses) or other personal beliefs. Methods: We retrospectively reviewed 490 patients who underwent either mastectomy alone (MA) or M-DTI between January 2021 and June 2023. We analyzed patient characteristics, operative details, and rates of bleeding complications. Results: MA was performed on 220 patients, while mastectomy with immediate breast reconstruction was conducted on 270 patients. Overall, bleeding complications—including those requiring reoperation and/or blood transfusion—occurred in 21 patients. In the MA group, bleeding complications occurred in 2.4% of patients, compared to 1.8% in the M-DTI group (*p* = 0.249). The risk of severe bleeding was unrelated to age, diagnosis, or type of procedure. Conclusions: The findings of this study suggest that immediate breast reconstruction does not increase the risk of bleeding in patients undergoing mastectomy. Therefore, it should also be considered a viable option for patients requiring bloodless medicine.

## Simple Summary

Immediate breast reconstruction has become a standard procedure that significantly improves the quality of life of patients after mastectomy without affecting oncological results. However, there are still doubts about whether to perform reconstructive surgery on patients who do not agree to a blood transfusion. This study aimed to evaluate whether direct-to-implant breast reconstruction poses a greater risk of bleeding complications than mastectomy alone and, therefore, is a viable option for bloodless medicine patients. The study results may help to establish standards of care for bloodless patients.

## 1. Introduction

Breast cancer is the most prevalent cancer among women globally, with over 2 million cases reported each year [1]. According to the Polish Cancer Registry, approximately 20,000 cases and 7000 deaths are documented annually in Poland [2]. The diagnosis and treatment of breast cancer significantly affect women’s physical and emotional well-being, as well as their self-perception of body image [3,4,5].

Although breast-conserving surgery (BCS) is the preferred approach in early breast cancer patients, mastectomy is carried out on patients who do not meet the criteria for BCS. It is recommended for patients with multifocal disease, a higher tumour stage, or when the anticipated cosmetic outcome is inadequate [6,7]. It also plays a significant role in genetically predisposed patients (e.g., BRCA1 or BRCA2 mutation carriers), considering risk-reducing mastectomy (RRM) [8].

Breast reconstruction has become an integral part of treatment, demonstrating that it is both safe and feasible during oncological therapy, without compromising treatment outcomes [9,10]. Recent studies have strongly proved that immediate breast reconstruction (IBR) improves self-image, enhances sexual health, and boosts psychosocial well-being [11,12,13,14,15,16]. As a result, in the era of heightened awareness of the patients, the number of immediate breast reconstructions has seen a significant increase [17].

Although breast cancer surgery is associated with a low rate of morbidity, the potential complications are numerous and may occur with different severities and presentations. They may include seroma, surgical site infection, hematoma, wound dehiscence, skin necrosis, lymphedema, and chronic postoperative pain [18,19,20,21]. Clinically significant bleeding is a rare complication; however, when it does occur, it may necessitate surgical revision, potentially resulting in the need for blood transfusion. [22].

The rejection of whole blood or component blood product transfusions is primarily a concern for Jehovah’s Witnesses (JW). Jehovah’s Witnesses are a Christian denomination with approximately 8.7 million members worldwide and 115,000 members in Poland, representing a significant social group. JW members not only decline whole blood and its main components but also refuse to donate or store their blood for transfusion purposes. Operating on such a patient presents a challenge for the surgeon and requires establishing an appropriate protocol [23,24].

The study aimed to compare the frequency of postoperative bleeding complications (hematomas requiring reoperation and/or blood transfusion) in patients undergoing mastectomy alone (MA) vs. mastectomy with immediate breast reconstruction (M-DTI) and to establish whether the IBR increases the risk and is consequently a safe and feasible procedure for bloodless patients.

## 2. Materials and Methods

A total of 490 patients who underwent mastectomy (either alone or with direct-to-implant immediate breast reconstruction) between January 2021 and June 2023 were included in this retrospective study. The study received approval from the Ethical Committee at the Polish Mother’s Memorial Hospital in Łódź (protocol no. 22/2022; approved date 15 March 2022).

The study included patients scheduled for mastectomy regardless of the diagnosis. Two hundred twenty patients underwent mastectomy without reconstruction, while 270 patients had mastectomy with implant-based immediate breast reconstruction. Table 1 presents the patients’ characteristics.

Before the operation, all patients were informed about the scheduled procedure and its associated risks, including the potential need for a blood transfusion. Patients who did not consent to transfusion indicated this in their consent to the procedure or provided their document of non-consent. We collected data on patient age, diagnosis, staging (for cancer patients), operation details, and complication rates.

All the patients received thromboprophylaxis one day before the procedure. Low molecular weight heparin (LMWH) was prescribed at 8 pm the night before the surgery and administered subcutaneously in a single dose of 40 mg. Patients receiving anticoagulants were prepared for the procedure according to the protocols. Patients receiving antiplatelet drugs and vitamin K antagonists had their treatment discontinued 5 days before the surgery. Direct oral anticoagulation was discontinued 2 days before the surgery.

All the operations (both MA and M-DTI) were performed with the use of electrocautery and bipolar forceps for hemostasis. All the immediate breast reconstructions were prepectoral, with the use of anatomical implants and synthetic meshes in one of two types—non-absorbable or absorbable.

The two groups were compared regarding bleeding complications, including hematoma requiring reoperation, blood transfusions, or both. Indications for the reoperation included expanding hematoma with jeopardized skin flaps or hemodynamic instability. Blood transfusion was scheduled when the hemoglobin level was less than 7 g/dL or 7.5 g/dL with accompanying symptoms of anemia (tachycardia, low blood pressure, dyspnea) [25].

Categorical variables were presented as numbers and percentages. Continuous variables were assessed for normality using the Shapiro–Wilk test. For variables not following a normal distribution, comparisons between the bleeding and non-bleeding groups were performed using the Mann–Whitney U test. Box-and-whisker plots were used to graphically present the comparisons, with the median indicated by a small square, the interquartile range (IQR) by a larger box, and the minimum and maximum values represented by the whiskers. The influence of clinical and laboratory factors on the occurrence of bleeding was assessed using univariable and multivariable logistic regression. A *p*-value < 0.05 was considered statistically significant.

## 3. Results

**Figure 1:**
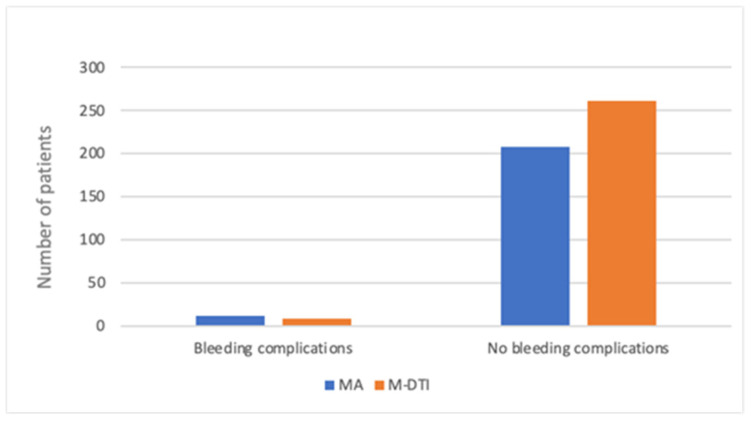
The frequency of bleeding complications in mastectomy alone (MA) and mastectomy with direct-to-implant immediate breast reconstruction (M-DTI) groups.

A total of 490 patients underwent mastectomy during the period above. M-DTI was performed in 270 patients (55.1%), while MA was performed in 220 patients (44.9%). Bleeding complications, which included both reoperation and blood transfusion, occurred in 21 patients overall (4.3%): 12 patients undergoing mastectomy alone, and 9—mastectomy with immediate breast reconstruction (2.4% for MA vs. 1.8% for M-DTI)—see Figure 1. There were no significant differences between the two groups (*p* = 0.249).

Univariable and multivariable logistic regression analyses assessing the association between clinical variables and bleeding events are presented in Table 2 and Table 3.

### 3.1. Age

**Figure 2:**
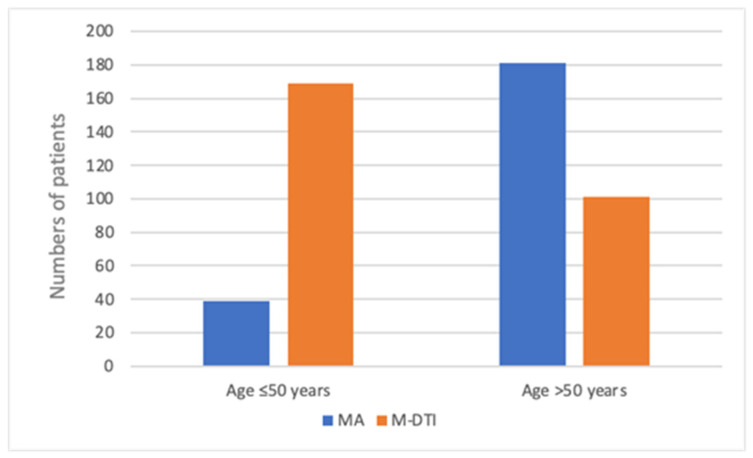
Age and type of procedure.

The mean age of the patients was 54 years (range, 24–92). Breast reconstruction was closely related to the patient’s age. In patients under 50 years, IBR was conducted in 81.3% (*n* = 169). Meanwhile, 64.2% of the patients over 50 years (*n* = 181) underwent mastectomy alone (Figure 2). Patients undergoing mastectomy alone were noted to be older, with a mean age of 69 years, compared to 51.5 years in the M-DTI group. There was no difference in the frequency of bleeding complications based on age (*p =* 0.188).

### 3.2. Diagnosis and Type of Procedure

A total of 73.9% of mastectomies (*n* = 411) were performed due to breast cancer (either invasive or in situ), 25.6% (*n* = 143) were carried out as risk-reducing procedures for high-risk mutation carriers, and 0.9% (*n* = 5) were conducted for other reasons, such as atypical granular cell tumour, phyllodes tumour, breast sarcoma, or breast papilloma.

The type of procedure (MA vs. M-DTI) was correlated with the diagnosis. In breast cancer patients, the results were comparable: 52.8% of these patients underwent mastectomy alone, whereas 47.2% underwent mastectomy with IBR. In the RRM group, 4.89% did not undergo breast reconstruction.

**Figure 3:**
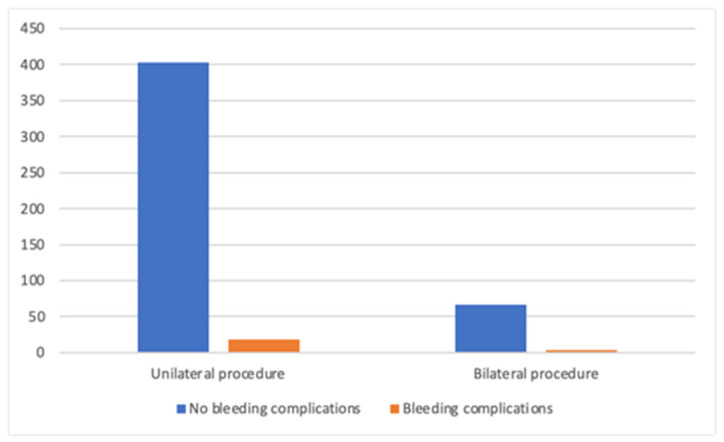
Bleeding complications in patients undergoing uni- and bilateral procedures.

**Figure 4:**
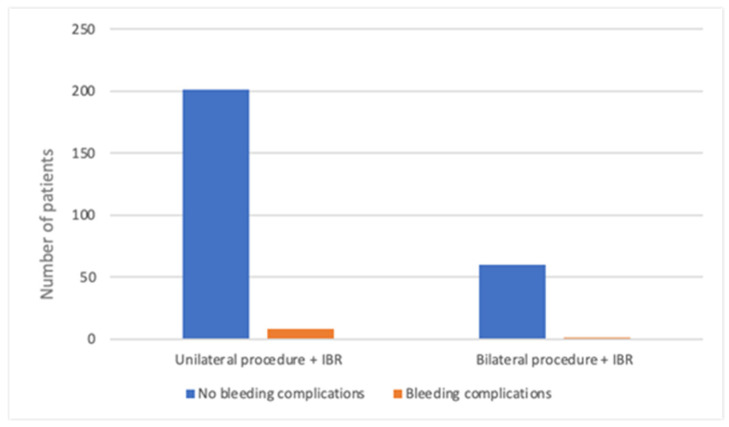
Bleeding complications in patients undergoing uni- and bilateral procedures with IBR.

Overall, 69 patients (14.1%) underwent bilateral procedures. In the MA group, 3.6% of patients (*n* = 8) underwent the bilateral procedure, compared to 22.6% (*n* = 61) in the M-DTI group. The increased number of contralateral prophylactic mastectomies in the M-DTI group accounts for the observed difference. The frequency of bleeding complications was similar, regardless of the extent of the procedure. In the entire analyzed population, bleeding complications occurred in 4% of patients in both groups (18/421 unilateral and 3/69 bilateral procedures; *p* = 0.134), as shown in Figure 3. In the M-DTI group, performing a bilateral operation did not increase the frequency of bleeding complications either (4% in unilateral vs. 2% in bilateral group; *p* = 0.402), as shown in Figure 4.

**Figure 5:**
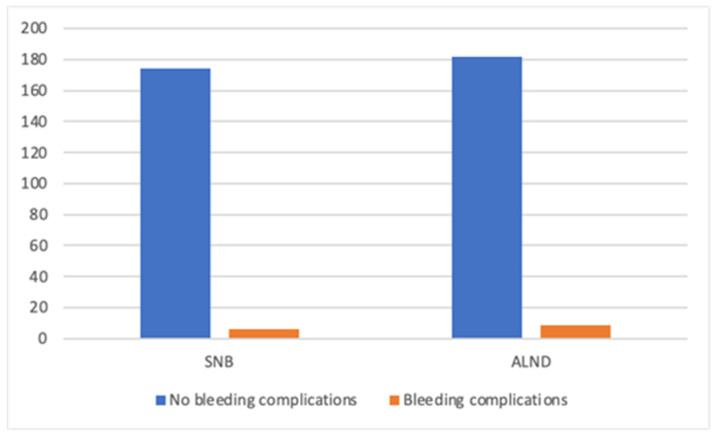
Bleeding complications in patients undergoing axillary surgery (SNB-sentinel lymph node biopsy, ALND-axillary lymph node dissection).

A total of 411 mastectomies were performed on patients diagnosed with breast cancer. In both groups, most patients presented with early-stage disease (Tis, T1, or T2). Bleeding complications were not dependent on the staging (*p =* 0.082). Alongside the mastectomy, breast cancer patients underwent an axillary procedure—either a sentinel lymph node biopsy (SNB) or an axillary lymph node dissection (ALND). ALND was carried out more frequently in patients undergoing mastectomy alone, 56% compared to 20.8% in the M-DTI group. The extent of the axillary procedure was not associated with bleeding complications (*p* = 0.500)—see Figure 5.

**Figure 6:**
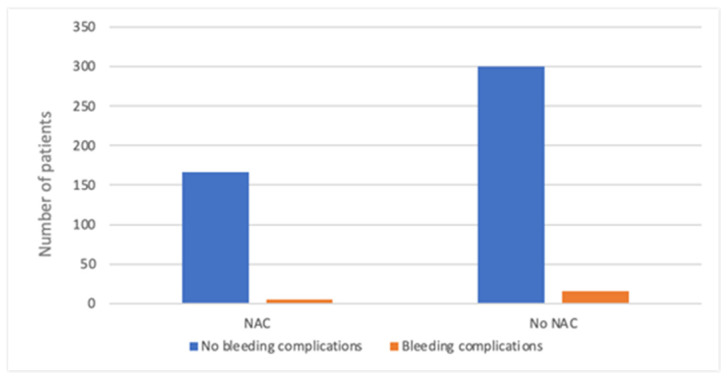
Bleeding complications in patients receiving neoadjuvant chemotherapy (NAC) and with no previous treatment.

Among the breast cancer patients, 35.7% (*n* = 175) received neoadjuvant chemotherapy. Preoperative treatment was more prevalent in the MA group—40.5% (*n* = 89) compared to 31.9% (*n* = 86) in the M-DTI group. There was no significant difference in bleeding complications between the group undergoing neoadjuvant chemotherapy compared to those with no prior treatment (*p* = 0.978) in Figure 6.

### 3.3. Patient-Related Factors

**Figure 7:**
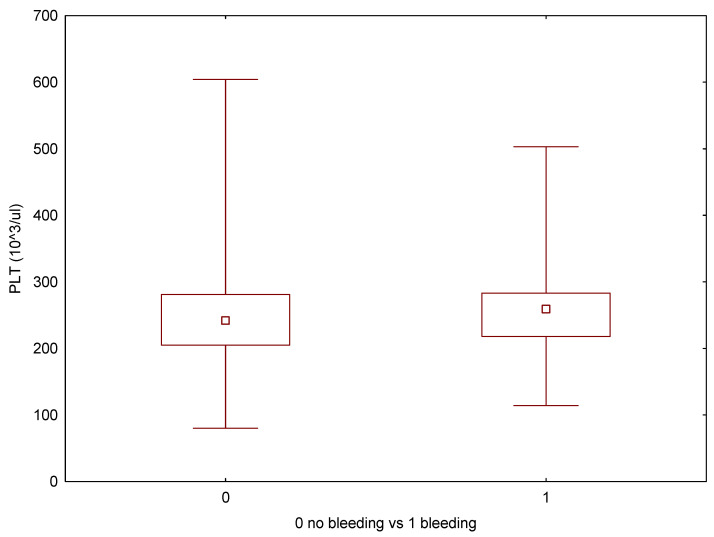
Box-whiskers plot comparing PLT levels between patients with and without bleeding events.

**Figure 8:**
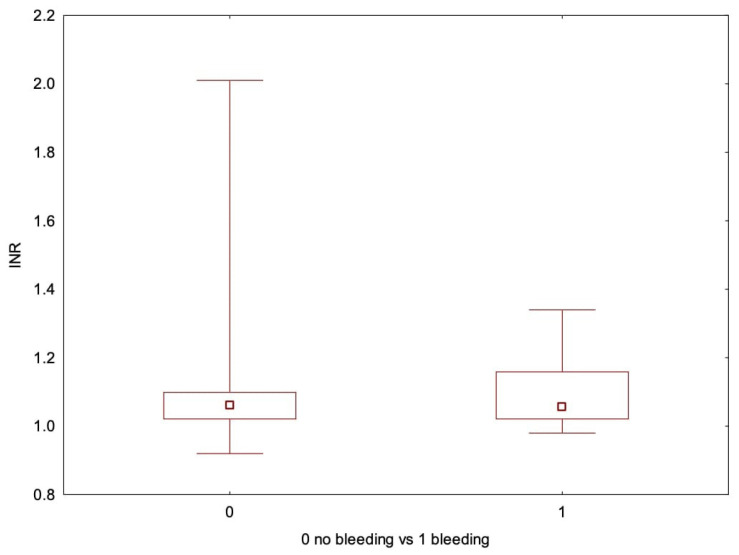
Box-whiskers plot comparing INR levels between patients with and without bleeding events.

**Figure 9:**
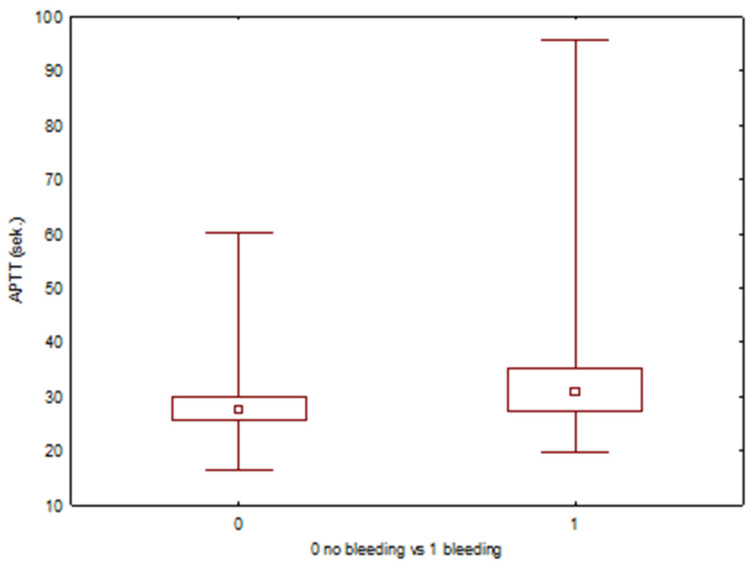
Box-whiskers plot comparing APTT levels between patients with and without bleeding events.

None of the patients had a prior diagnosis of a bleeding disorder. All patients receiving anticoagulants or antiplatelet medications had their treatment either discontinued or adjusted following current recommendations. We reviewed the preoperative blood test results for any irregularities to assess the patient’s related risk. (Table 4) 8.8% (*n* = 43) of patients exhibited incorrect coagulation parameters (PLT, INR, and/or APTT). Two patients with elevated APTT tested positive for lupus anticoagulant (LA dRVVT+). Box-and-whisker plots were used to graphically present the comparisons regarding PLT (Figure 7), INR (Figure 8), and APTT levels (Figure 9). In univariable logistic regression, APTT was significantly associated with an increased risk of bleeding (OR 1.14, 95% CI: 1.05–1.24, *p* = 0.001). In multivariable logistic regression analysis, APTT remained a significant predictor of bleeding risk (OR 1.10, 95% CI: 1.01–1.20, *p* = 0.033).

## 4. Discussion

The importance of breast reconstruction is highlighted by the number of studies and reports on patients’ quality of life following mastectomy. The removal of the breast, an organ symbolizing femininity, is associated with disturbances in body image. Given the high survival rates for breast cancer patients, greater emphasis should be placed on overall well-being, including mental health and sexuality [13,26]. Our study reveals a high need for M-DTI among younger patients, as this procedure was performed in 81.3% of patients under 50 years old. Numerous studies demonstrate, however, that due to the safety and benefits of this procedure, older age should not be a contraindication itself [27,28].

In our study, we did not observe a statistical difference in the frequency of bleeding complications depending on the type of procedure: it occurred in 2.4% of patients undergoing mastectomy alone and 1.8% of patients after immediate breast reconstruction. The study results suggest that oncological safety should be the main factor in deciding on breast reconstruction. The literature supports this finding. Complication rates in breast surgery vary across the literature in frequency and severity, and bleeding or hematoma is not the most common complication. According to Thalji et al., the incidence of postoperative hematoma requiring operative evacuation after breast surgery is estimated to be less than 2% and appears to be higher among patients receiving mastectomy alone compared to those undergoing immediate reconstruction or breast-conserving surgery [29]. Similar findings were reported by Abbate et al. in the meta-analysis of prepectoral implant-based breast reconstruction, where the incidence of hematoma was estimated at 1.86% [30]. The lower rate of hematomas after reconstruction remains unclear; it may be related to a different surgical technique, e.g., more frequent use of tumescent solution in M-DTI, as a means of facilitating dissection in the appropriate plane and lessening the intraoperative blood loss [30]. The impact of meshes and matrices on hematomas in M-DTI is also unknown, necessitating further prospective studies in that matter.

A multidisciplinary team that provides thorough preoperative preparation and meticulous perioperative management can ensure that surgery in bloodless patients is conducted without serious complications. Factors such as age, body mass index (BMI), comorbidities, the American Society of Anesthesiologists (ASA) classification, medications, and the type of procedure should be considered to assess the risk of surgery. Surgeons can utilize predictive tools, such as the Breast Cancer Surgery Risk Calculator (BCSR), to estimate the individual risk of complications [31]. Minimizing the risk of bleeding or anemia can be performed in advance. It is also possible to preoperatively correct existing anemia without the need for a blood transfusion. Once anemia is detected, a treatment that stimulates erythropoiesis and improves oxygenation should be implemented, such as intravenous iron or erythropoietin (EPO) administration [32,33]. In patients taking anticoagulants, it is necessary to discontinue them or apply bridging therapy. Abnormal coagulation parameters may also be a predictive factor in bleeding. In our study, the difference in APTT level between the group with and without bleeding complications was significant in the Mann–Whitney U test. Still, given the quantitative differences between the groups, this may affect the power of the test, constituting rather an exploratory result.

The next step is to minimize blood loss during the procedure, which can be achieved by using appropriate electrocautery or bipolar hemostatic forceps settings. Studies also indicate the potential for intravenous administration of tranexamic acid, which safely reduces the risk of hematoma in implant-based breast reconstruction without increasing the risk of thromboembolic events [34].

Although breast reconstruction has become a standard procedure, there is limited literature addressing this issue in bloodless patients. Larcher et al. described a successful DIEP (Deep Inferior Epigastric Perforator) flap reconstruction in a JW patient; however, they placed great emphasis on the surgeon’s consent to the procedure and the need for a comprehensive discussion about potential complications, including the risk of death from bleeding [35]. Other studies on reconstructive surgery have found that free flap procedures can be safely performed on JW patients, even without blood transfusions, provided that proper preoperative preparation and an adequate bloodless protocol are followed, which are key to ensuring good outcomes [36,37].

Although Jehovah’s Witnesses are primarily known for their opposition to blood transfusions, the benefits of abstaining from blood products are increasingly being highlighted. Numerous studies indicate that blood transfusion is an independent risk factor for increased morbidity [38]. Current guidelines regarding blood transfusions in cancer patients are stringent, as the rapid but temporary effect may not outweigh the risks of post-transfusion complications, viral infections, allergic reactions, or secondary immunosuppression. The cytokines released through the transfusion of blood components (including interleukins IL-6, IL-8, and IL-10) have exhibited pro-inflammatory and immunosuppressive effects, and their clinically significant interaction with the mechanisms of action of drugs that modulate the immune system, used in oncology, cannot be overlooked [39]. While the controversy over the lack of consent for transfusion persists, careful consideration for using blood components appears to be warranted.

Most blood-declining patients are members of the Jehovah’s Witness faith. Jehovah’s Witnesses typically carry a legally binding document stating that they should not be given any blood products in their treatment under any circumstances. Although medical laws vary worldwide, most European countries emphasize obtaining informed consent from patients for any medical procedure. In Poland, patients’ rights are regulated by the Polish Constitution and various legal acts, including the Act on the Profession of Doctor and Dentist, the Act on Patients’ Rights, and the Act on the Patient’s Rights Advocate. To adhere to the legal doctrine of informed consent, physicians must respect the personal autonomy and decision-making abilities of competent patients [40,41,42,43]. Any medical procedure, including transfusions of whole blood or blood components, carried out against the patient’s will, violates their autonomy, which has been upheld in the Polish judiciary, affirming that the absence of consent for a specific procedure is binding on the physician and eliminates any criminal or civil liability [44]. Another significant legal aspect is the physician’s ability to withdraw from therapy unless the patient’s condition is urgent [45]. Surgeons should always discuss potential complications with the patient, as this can enhance the patient-physician decision-making process.

This study’s limitations include its retrospective nature and non-randomized design, which may lead to selection bias. Breast reconstruction was strictly related to age; younger, healthier patients opted for M-DTI. In contrast, the median age of patients eligible for MA was significantly higher, and these patients often had more advanced-stage disease. We also noted that our data did not consider the proportion of patients who did not consent to blood transfusion. To accurately assess this risk, prospective validation is necessary, along with a comprehensive evaluation of patient-related factors such as BMI, existing comorbidities, and ASA score. Our study, however, aimed to highlight the existing challenges associated with blood transfusions in underrepresented patient groups and to promote patient-centred care.

## 5. Conclusions

In conclusion, implant-based immediate breast reconstruction after mastectomy does not increase the risk of bleeding complications. A decline in blood transfusions should not dictate the use of IBR, particularly given the improvement in postoperative quality of life and the absence of interference with oncological treatment. Unless there are oncological contraindications, breast reconstruction should be offered to all patients scheduled for mastectomy. With adequate preparation, a personalized approach, and evaluation of risk factors, we propose that IBR is a safe and viable procedure to enhance outcomes. Techniques developed in the care of JW patients should be widely utilized to improve results and reduce healthcare costs.

## Figures and Tables

**Table 1:** Patients’ characteristics.

	Mastectomy Alone—MA Group (*n* = 220)	Mastectomy with Direct-to-Implant Breast Reconstruction—M-DTI Group (*n* = 270)	Total (*n* = 490)
Age (mean)	69	51.5	54
Range	28–92	24–76	24–92
Age, years			
≤50	39 (17.7)	169 (62.6)	208 (42.4)
>50	181 (82.3)	101 (37.4)	282 (57.6)
Mastectomy type			
Unilateral	212 (96.4)	209 (77.4)	421 (85.9)
Bilateral	8 (3.6)	61 (22.6)	69 (14.1)
Diagnosis (per breast)	*n* = 227	*n* = 332	*n* = 559
Breast cancer	217 (95.6)	194 (58.4)	411 (73.5)
T is/1/2	184 (81.1)	187 (56.3)	371 (66.4)
T 3/4	33 (14.5)	6 (1.8)	40 (7.2)
RRM	7 (3.1)	136 (41.0)	143 (25.6)
Other	3 (1.3)	2 (0.6)	5 (0.9)
Axillary surgery			
None	25 (11.0)	149 (44.9)	174 (31.1)
SNB	75 (33.0)	114 (34.3)	189 (33.8)
ALND	127 (56.0)	69 (20.8)	196 (35.1)
NAC	89 (40.5)	86 (31.9)	175 (35.7)
Bleeding disorder			
	25 (11.4)	18 (6.7)	43 (8.8)
PLT < 150 × 10^9^/L	12	9	21
INR > 1.2	8	5	13
APTT > 36.9	8	4	12

Data are presented as *n* (%) unless otherwise noted; RRM—risk-reducing mastectomy, NAC—neoadjuvant chemotherapy, SNB—sentinel node biopsy, ALND—axillary lymph node dissection, PLT—platelets, INR—International Normalized Ratio, APTT—Activated Partial Thromboplastin Time.

**Table 2:** Univariable Logistic Regression Evaluating the Association Between Clinical Variables and Bleeding Events.

Variable	OR	95% CI Lower	95% CI Upper	*p*-Value
Age	1.01	0.98	1.04	0.389
M-DTI	0.67	0.28	1.57	0.355
Bilateral surgery	0.96	0.28	3.34	0.951
Bilateral surgery + IBR	0.37	0.05	2.98	0.351
Neoadjuvant chemotherapy	0.53	0.19	1.46	0.220
ALND (vs SNB)	1.60	0.57	4.50	0.371
PLT [G/l]	1.00	0.99	1.01	0.882
INR	27.18	0.87	849.57	0.060
APTT [s]	1.14	1.05	1.24	0.001

OR—odds ratio, CI—confidence intervals, IBR—immediate breast reconstruction, SNB—sentinel node biopsy, ALND—axillary lymph node dissection, PLT—platelets, INR—International Normalized Ratio, APTT—Activated Partial Thromboplastin Time.

**Table 3:** Multivariable Logistic Regression Evaluating the Association Between Clinical Variables and Bleeding Events.

Variable	OR	95% CI Lower	95% CI Upper	*p*-Value
Age	1.00	0.96	1.05	0.989
PLT [G/l]	1.00	0.99	1.01	0.849
INR	9.59	0.18	514.69	0.266
APTT [s]	1.10	1.01	1.20	0.033
M-DTI	0.83	0.22	3.18	0.786
Bilateral surgery	2.31	0.44	12.18	0.323
Neoadjuvant chemotherapy	0.54	0.16	1.79	0.316
ALND (vs SNB)	1.66	0.53	5.22	0.386

OR—odds ratio, CI—confidence intervals, IBR—immediate breast reconstruction, SNB—sentinel node biopsy, ALND—axillary lymph node dissection, PLT—platelets, INR—International Normalized Ratio, APTT—Activated Partial Thromboplastin Time.

**Table 4:** Patients’ coagulation parameters.

	Min	Max	Average	Median	SD	Average—Bleeding Complications	Average—No Bleeding Complications	*p* (U-Mann–Whitney)
PLT (10^3^/μL)	80	604	246	242,5	64.93	245	247	0.704
INR	0.92	2.01	1.06	1.06	0.079	1.09	1.06	0.200
APTT (s)	16.6	95.6	28.39	27.6	4.91	34.34	28.12	0.002

## Data Availability

The research data are available upon request after direct contact with the corresponding author.

## Abbreviations

The following abbreviations are used in this manuscript:

M-DTI	Mastectomy with direct-to-implant reconstruction
MA	Mastectomy alone
JW	Jehovah’s Witnesses
BCS	Breast Conserving Surgery
IBR	Immediate Breast Reconstruction
RRM	Risk Reducing Mastectomy
SNB	Sentinel Node Biopsy
ALND	Axillary Lymph Nodes Dissection
NAC	Neoadjuvant Chemotherapy
PLT	Platelets
APTT	Activated Partial Thromboplastin Time
INR	International Normalized Ratio
OR	Odds ratio
CIs	Confidence intervals
LMWH	Low molecular weight heparin
BMI	Body Mass Index
EPO	Erythropoietin
ASA	American Society of Anesthesiologists
BCSR	Breast Cancer Surgery Risk
